# Hybrid Clustered Nanoparticles for Chemo-Antibacterial Combinatorial Cancer Therapy

**DOI:** 10.3390/cancers11091338

**Published:** 2019-09-10

**Authors:** Barbara Cortese, Stefania D’Amone, Mariangela Testini, Patrizia Ratano, Ilaria Elena Palamà

**Affiliations:** 1 Nanotechnology Institute, CNR-NANOTEC, University La Sapienza, P.zle A. Moro, 00185 Rome, Italy; 2 Nanotechnology Institute, CNR-NANOTEC, via Monteroni, 73100 Lecce, Italy

**Keywords:** nanoparticles, combinatorial therapy, anticancer and antibacterial activity

## Abstract

*Background*: A great number of therapeutic limitations, such as chemoresistance, high dosage, and long treatments, are still present in cancer therapy, and are often followed by side effects such as infections, which represent the primary cause of death among patients. *Methods*: We report pH- and enzymatic-responsive hybrid clustered nanoparticles (HC-NPs), composed of a PCL polymeric core loaded with an anticancer drug, such as Imatinib Mesylate (IM), and coated with biodegradable multilayers embedded with antibacterial and anticancer baby-ship silver NPs, as well as a monoclonal antibody for specific targeting of cancer cells conjugated on the surface. *Results*: The HC-NPs presented an onion-like structure that serially responded to endogenous stimuli. After internalization into targeted cancer cells, the clustered nanoparticles were able to break up, thanks to intracellular proteases which degraded the biodegradable multilayers and allowed the release of the baby-ship NPs and the IM loaded within the pH-sensible polymer present inside the mothership core. In vitro studies validated the efficiency of HC-NPs in human chronic leukemic cells. This cellular model allowed us to demonstrate specificity and molecular targeting sensitivity, achieved by using a combinatorial approach inside a single nano-platform, instead of free administrations. The combinatory effect of chemotherapic drug and AgNPs in one single nanosystem showed an improved cell death efficacy. In addition, HC-NPs showed a good antibacterial capacity on Gram-negative and Gram-positive bacteria. *Conclusions*: This study shows an important combinatorial anticancer and antimicrobial effect in vitro.

## 1. Introduction

Cancer represents one of the most formidable reasons of death in the world, despite important advances in oncology. Clinical practice indicates that cytotoxic therapeutic molecules are most effective when provided in combination, in order to realize synergistic or additive outcomes [1,2], even though important side effects [3], such as cardiotoxicity and bone marrow suppression [4,5], are reported. An underestimated important consequence of chemotherapy is represented by infections after treatment that remain the main cause of hospitalization and death amongst most patients [6]. Combinatorial cancer therapy, with the aim to prevent infection and to specifically kill cancer cells, can result in important cutbacks in morbidity and mortality for cancer patients. Recent progress in nano-cancer therapy represents an important oncological challenge [7]. Nanomedical materials have improved the gold standard in drug delivery with regards to biodistribution, intracellular uptake, and dosing efficacy, thanks to the use of nanoparticles (NPs) that are able to load therapeutic agents and specifically target the disease [8]. Currently, different liposome- and polymer-based NPs have been approved by the Food and Drug Administration (FDA) for clinical use [7].

In addition, the development of multifunctional materials for combination therapy via nanoparticles has emerged as an innovative therapeutic stratagem to attain synergistic combination effects for cancer therapy [9]. Multifunctional NPs have the ability to deliver different therapeutic agents and to improve drug efficiency within single administrations, releasing drug at the action site and reducing side effects [7,10,11,12]. Recent work has reported that the use of NPs with anti-cancer properties and chemotherapic drugs for combinatorial cancer therapy have shown an improved efficacy of chemotherapy, minimizing toxic side effects [13,14,15].

In this context, inorganic nanoparticles [15], such as silver nanoparticles (AgNPs), have shown important cancer activity [14,16,17], and numerous studies have described the cytotoxicity of AgNPs as being accredited to their ability to release Ag ions in the lysosome acid environment, inducing reactive oxygen species (ROS) production, leading to DNA damage and cell death [18,19,20]. In addition, the use of AgNPs as an antimicrobial agent in humans, has been approved by the FDA in situations such as in burns and wound healing [21].

Recently, we have shown how a combination of two different drugs loaded inside one nanosystem, such as Poly-caprolactone (PCL) NPs, can be used to overcome the problem of drug resistance, with low concentrations and showing a synergic effect [10].

In this paper, we move forward from our previous results, proposing to overcome actual clinical therapeutic problems associated with a high dose of chemotherapic drugs and infections, by developing hybrid clustered nanoparticles (HC-NPs), which can show combined chemo/antibacterial potential and therapeutic advantages in order to reach a cancer cure, and in reducing adverse effects such as infections. The chemo/antibacterial potential of our HC-NPs were investigated on chronic myeloid leukemia (CML), used as a model of cancer. CML is a stem cell-derived disorder [22], generated by a BCR-ABL oncoprotein. The current gold standard therapy is represented by tyrosine kinase inhibitors (TKIs), such as imatinib mesylate (IM). In addition, leukemia stops the bone marrow from producing enough healthy white blood cells, thus the CML patient is subject to infections. 

Our HC-NPs presented an onion-like structure which was composed of compartmentalized loaded payload polymeric nanovectors functionalized with specific monoclonal antibody (mAb), in order to specifically target cancer and simultaneously prevent bacterial infections.

Specifically, our nanosystem was composed of two different nanoparticles: a mothership nanocarrier (size of approximately 200 nm) which represented the inner core, and 30 nm-sized babyship nanocarriers attached to the surface of the mothership. The mothership nanocarrier was produced using FDA-approved PCL polymer and was loaded with IM [10] in order to kill leukemia cells. Babyship nanoparticles were composed of silver in order to induce anticancer and antibacterial effects.

In vitro studies showed excellent anti-leukemic activity of our HC-NPs, thanks to the combinatory effect of IM and AgNPs that improved cell death efficacy. In addition, our HC-NPs showed a good antibacterial capacity on Gram-negative and Gram-positive bacteria. 

To the best of the authors’ knowledge, this is the first time that a chemo-antibacterial combinatorial therapy strategy has been developed, which potentiates single drugs and elicits an antibacterial response. Our strategy may have important therapeutic and pharmacological applications in cancer therapy, in order to simultaneously kill cancer cells and prevent infections.

## 2. Results

### 2.1. Synthesis and Characterization of Hybrid Cluster NPs

**Figure 1:**
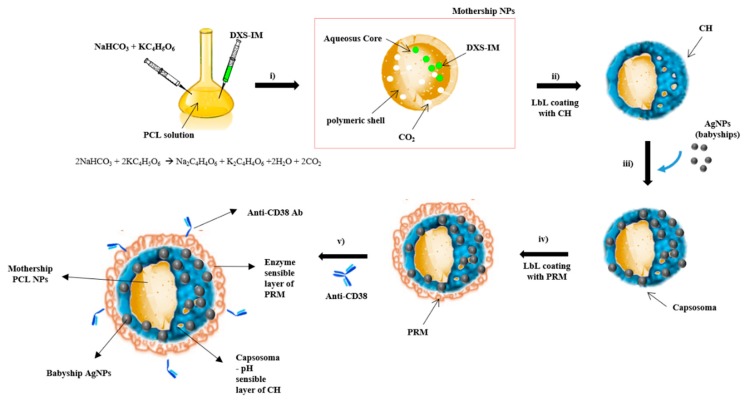
Illustration of the production of HC-NPs. AgNPs: silver nanoparticles, PRM: enzyme sensible protamine, PCL: Poly-caprolactone polymer

Our HC-NPs presented an onion-like structure (Figure 1) in which two different types of NPs were combined in order to improve the therapeutic efficacy. HC-NPs were composed of (i) a mothership biodegradable polymeric inner core (PCL), loaded with IM; (ii) an intermediate layer (capsosoma) composed of pH-sensible chitosan (CH) filled with babyship AgNPs; and (iii) a final outer layer (enzyme sensible protamine, PRM) functionalized with a specific monoclonal antibody, an anti-CD38 [23] antibody (Ab), for cell targeting. The synthesis of hybrid cluster NPs (HC-NPs) are illustrated in Figure 1.

Prior to assemblage of HC-NPs, the silver (babyship) and PCL NPs (mothership) were synthetized separately and characterized.

Colloidal suspension of silver nanoparticles (AgNPs) were prepared by reduction of an aqueous solution containing AgNO_3_ with NaBH_4_. The color change of solution from transparent to yellow indicated the formation of AgNPs, also confirmed by UV-VIS spectroscopy (Supplementary Figure S1). After the synthesis, AgNPs were centrifuged and resuspended in sodium citrate solution. AgNPs were shown to have a mean size of about 32 nm with a zeta potential of −27.9 mV (Table 1). Transmission electron microscopy (TEM) analysis of AgNPs (Supplementary Figure S2A) showed a spherical morphology and no aggregation.

Core-shell IM-loaded PCL NPs were prepared by an emulsion–diffusion–evaporation modified method [10], which allowed for the obtaining of NPs with a size of about 228 nm and with a ζ-potential of –11 mV (Table 1; Supplementary Figure S2B). Before the encapsulation of IM into the hydrophilic core of PCL NPs, these were complexed with dextran (DXS), an enzyme-sensible polymer [24] that permitted their controlled release after degradation by intracellular protease.

Hybrid IM-PCL/Ag cluster NPs (HC-NPs) were obtained following different steps (Figure 1). In order to assemble babyship and mothership NPs to form a capsosoma, the surface of IM-PCL NPs (mothership NPs) were coated with the layer-by-layer technique (LbL) using chitosan, a pH-sensible polymer, that allows the initial release of AgNPs in a cytoplasmic environment (Figure 1ii). Subsequently, a suspension of AgNPs (babyship NPs) was added, and an additional outer layer, due to the electrostatic interactions, was formed onto the surface of mothership NPs (Supplementary Figure S2C), obtaining a capsosoma. Size and zeta potential modifications in the mothership NPs after deposition of babyship NPs’ layer were analyzed with Dynamic Light Scattering (DLS) (Table 1). Moreover, we measured using a DLS analysis the fixed aqueous layer thickness (FALT) [25,26] of our NPs (Table 1).

By adding a new layer on capsosoma, we observed an increase of the FALT value, showing that a fixed aqueous layer was formed on the surface of NPs. In addition, the assembly of HC-NPs was confirmed by FT-IR analysis (Supplementary Figure S3).

To provide the final outermost layer functionalized with anti-CD38 antibody, the capsosoma was coated with enzyme-sensible protamine (PRM) [24] solution (Supplementary Figure S2D) and a covalent binding of anti-CD38 antibody on the capsosoma surface was performed using EDC (1-ethyl-3-(3-dimethylaminopropyl)-carbodiimide). FACS (Fluorescence-activated cell sorting) analysis (Supplementary Figure S4A, red line) revealed that the HC-NPs were efficiently functionalized with the anti-CD38 antibody, and also that the integrity of the antibody was confirmed by SDS-PAGE, as shown in Supplementary Figure S4B.

The IM encapsulation efficiency (EE%) obtained in this study was of about 95% in PCL NPs and of 73% for AgNPs.

Release study of babyship AgNPs and IM loaded into the mothership core from HC-NPs was performed, mimicking the different intracellular compartments. In particular, HC-NPs were incubated in PBS at pH 5.8 and 7.4, in order to mimic the lyso/endosomal and cytoplasmatic environments, respectively. IM was released after degradation of protease-sensible DXS, and a release of about 42% was observed at pH 5.8 after 96 h (Supplementary Figure S5A).

For babyship AgNPs, we observed a biphasic release pattern (Supplementary Figure S5B). An initial burst release was followed by a sustained release at pH 5.8. A low release of about 5.5% was observed at pH 7.4 after 96 h. On the contrary, we observed an accelerated release of about 81% at pH 5.8, which seemed to be related to the presence of the combination of sodium bicarbonate and potassium tartrate in the core of the mothership NPs.

An IM release of about 23% and of about 32% for AgNps after 96 h at pH 5.8 was evident with control HC-NP formulations without the mixture of sodium bicarbonate and potassium tartrate in the mothership core, respectively (Supplementary Figure S5C,D).

In addition, HC-NPs maintained their hydrodynamic size and release profiles over a time window of 8 days when incubated in physiological conditions, and this evidence supports their stability. 

### 2.2. Protein Corona Analysis

**Figure 2:**
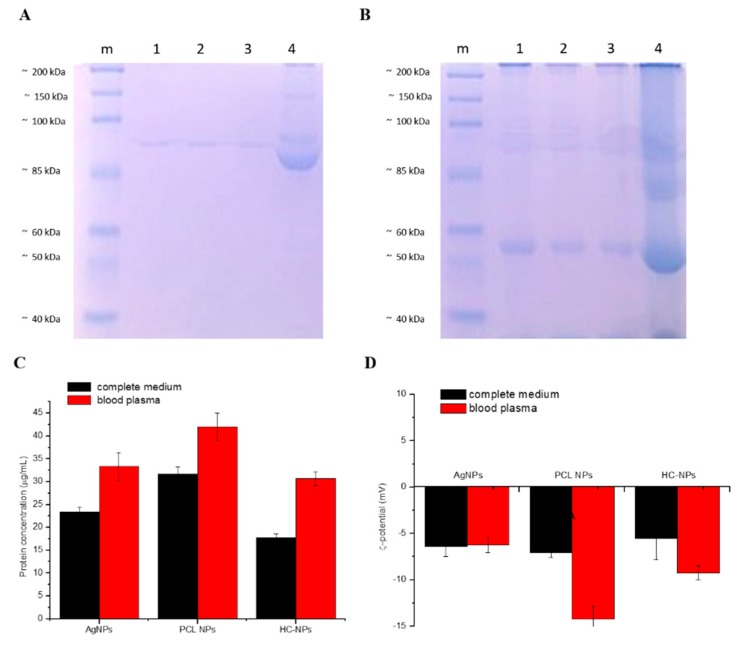
SDS-PAGE of hard protein corona after incubation with complete culture medium (**A**), and with mouse blood plasma (**B**) overnight at 37 °C with AgNPs (lane 1 of A and B); PCL NPs (lane 2 of A and B), HC-NPs (lane 3 of A and B), and control with only medium (lane 4 of A) and with solely mouse blood plasma (lane 4 of B). Quantification of adsorbed proteins on different NPs formulation by the bicinchoninic acid (BCA) assay (**C**). ζ-potential analysis of NPs after incubation with complete medium and blood plasma overnight (**D**). A representative result of three independent experiments is shown (*P*-values 0.05).

Our NPs (e.g., AgNPs, PCL NPs, and HC-NPs) were incubated with complete cell culture medium with 10% FBS or mouse blood plasma overnight in agitation at 37 °C. After the washing steps, the NPs’ hard corona was analyzed with SDS-PAGE. As shown in Figure 2A,B, the quantity of hard protein corona by incubation of complete medium and blood plasma on the surface of AgNPs and PCL NPs appeared to be higher (lanes 1, 2) compared to HC-NPs (lanes 3). This was confirmed by the data from the bicinchoninic acid (BCA) assay (Figure 2C). 

In addition, zeta-potential analysis after incubation in complete medium or mouse blood plasma of different formulations of NPs suggested that the adsorption of serum proteins induced a change in their zeta potential (Figure 2D), confirming the formation of protein corona around the NPs.

Hemocompatibility of our formulation was analyzed performing a hemolysis assay. As shown in the Supplementary Figure S6, the HR% of AgNPs and HC-NPs was lower compared to the positive control (physiological solution and 2% blood), and in contrast, the HR% of PCL NPs was slightly higher, but still negligible. 

### 2.3. Antibacterial Action

**Figure 3:**
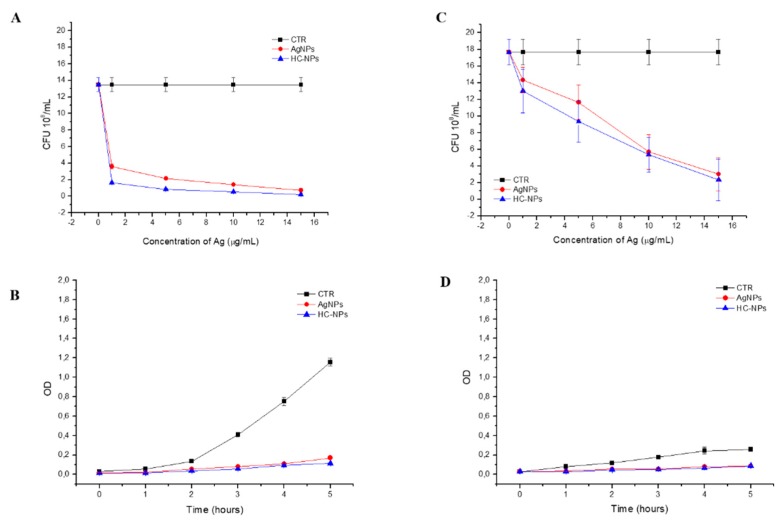
Antibacterial analysis on Gram-negative *E. coli* DH5α (**A**,**B**) and Gram-positive *S. aureus* (**C**,**D**) of AgNPs and HC-NPs by colony-forming units (CFU) counting on solid Luria–Bertani (LB) broth (**A**,**C**) as a function of Ag concentrations after 24 h. Measurement of optical density (OD) (**B**,**D**) as a function of time. Representative measurements of three different sets of data have been reported (*P*-values 0.05).

The antibacterial properties of the babyship AgNPs and HC-NPs were evaluated by measurement of bacteria growth in function of the optical density (OD, Figure 3B,D) and by the number of colony-forming units (CFU, Figure 3A,C) on Gram-negative *E. coli* DH5α and Gram-positive *S. aureus*. The antibacterial activity, reported in Figure 3, showed a good antibacterial capacity of the babyship AgNPs and HC-NPs on Gram-negative DH5α *E. coli*, and Gram-positive *S. aureus*, thus confirming that the babyship AgNPs inside the HC-NPs maintained the antibacterial efficacy, despite the assembly with the mothership NPs.

### 2.4. Targeting and Cellular Uptake Analysis

**Figure 4:**
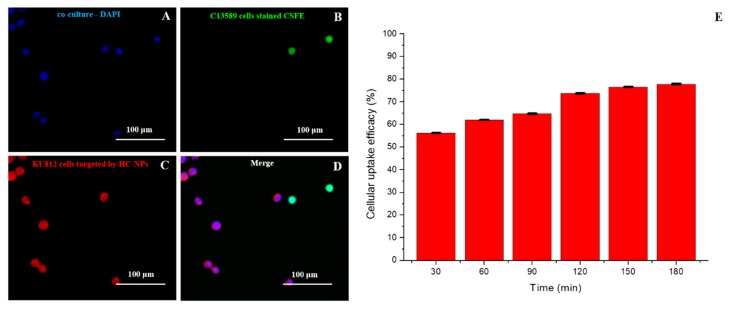
Confocal Laser Scanning Microscopy (CLSM) images of C13589 and KU812 cell co-culture. (**A**) Cell nuclei were counterstained with 4′,6-diamidino-2-phenylindole (DAPI,blue), (**B**) C13589 cells before of co-culture were stained with commercial CSFE (carboxyfluorescein diacetate, succinimidyl ester) fluorophore (green), (**C**) KU812 cells present in the co-culture were recognized by HC-NPs functionalized with anti-CD38 monoclonal antibody (red), (**D**) merged confocal images. Scale bars: 100 μm. Time-dependent cellular uptake efficiency of HC-NPs by KU812 cells (**E**). Representative measurements of three independent experiments have been reported (*P*-values 0.05).

Specific cell targeting of HC-NPs was analyzed in a co-culture of CD38-positive (KU812) and CD38-negative (C13895) cells after treatment with 500 ng/mL of Tetramethylrhodamine (TRITC)-conjugated anti-CD38 HC-NPs for 24 h. As shown in Figure 4, the antibody attached onto the HC-NPs surface can specifically target leukemia KU812 cells in a co-culture. The cellular uptake efficacy of HC-NPs was quantitatively evaluated by fluorimeter analysis, as shown in Figure 4E. 

The cellular uptake was time-dependent, and surface functionalization of HC-NPs with anti CD38 antibody revealed an improved cellular uptake for leukemia compared with other NP formulations that do not present the antibody on their surface (Supplementary Figure S7).

### 2.5. Anti-Cancer Efficacy of HC-NPs

The anti-cancer effect of HC-NPs was studied in vitro using different NP formulations. Free IM and empty PCL NPs were used as controls. No cytotoxicity on healthy C13895 cells was noticed using the IC_50_ doses of all formulations (Supplementary Figure S8), confirmed by cell cycle analysis, as shown in Supplementary Figure S9. On KU812 leukemia cells, we observed a dose-dependent effect (Supplementary Figure S10A) when the leukemia cells were incubated with all NP formulations (IM-loaded PCL NPs, AgNPs, and HC-NPs). In particular, HC-NP formulation showed an improved cytotoxicity.

In Supplementary Figure S10B, the IC_50_ values of different formulations on KU812 leukemia cells are shown, and it is possible to observe an IC_50_ value much lower for HC-NPs due to the controlled release of the drug and the AgNPs. In addition, a combination index (CI) of 0.81 of IM and AgNPs on KU812 leukemia cells showed a synergistic effect.

**Figure 5:**
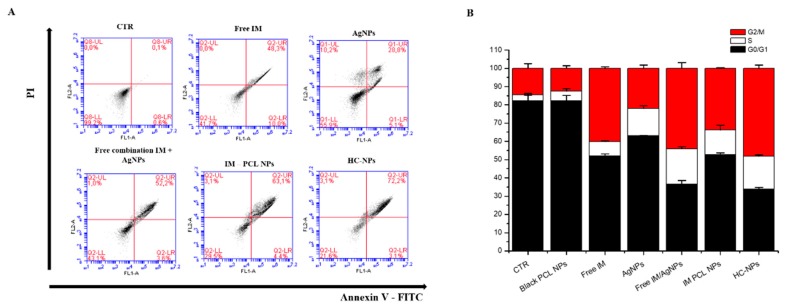
Analysis of cell apoptosis (**A**) and cell cycle (**B**) of KU812 leukaemia cells after 24 h of treatments with different formulations, compared with untreated control cells (CTR). Representative measurements of three independent experiments have been reported (*P* 0.05).

Combinatorial effect on KU812 leukaemia cells was analyzed through FACS analysis using annexin V-FITC/PI staining. Flow cytometry plots of leukemia KU812 cells (Figure 5A) indicated an evident apoptosis when incubated with HC-NPs (about 72%). In addition, cell cycle blocking at G2/M phase (see Figure 5B) was evident with treatment with HC-NPs (~45%).

**Figure 6:**
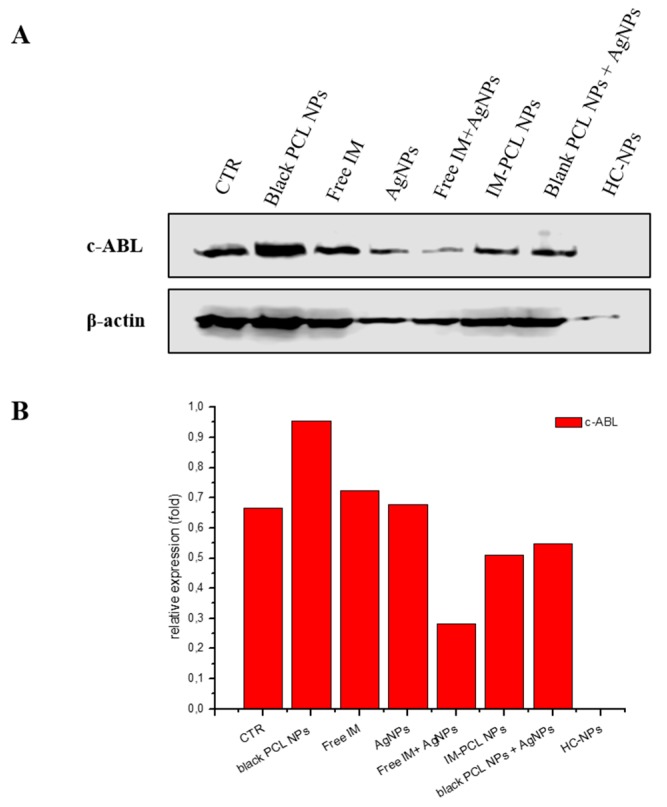
Inhibition of the oncoprotein BCR-ABL with western blotting after 7 days of treatment with different formulations (**A**) and densitometry analysis (**B**) of c-ABL levels normalized to the β-actin used as control. Representative measurements of three independent experiments have been reported.

The inhibition of tyrosine kinase activity of the oncoprotein BCR-ABL in leukemic cells KU812 was evaluated by western blotting analysis (Figure 6), after 7 days of incubation with the different formulations. Western blotting analysis was conducted using anti c-ABL antibody. As shown in Figure 6, a total inhibition of BCR-ABL was evident in treating the leukemia cells with HC-NPs.

**Figure 7:**
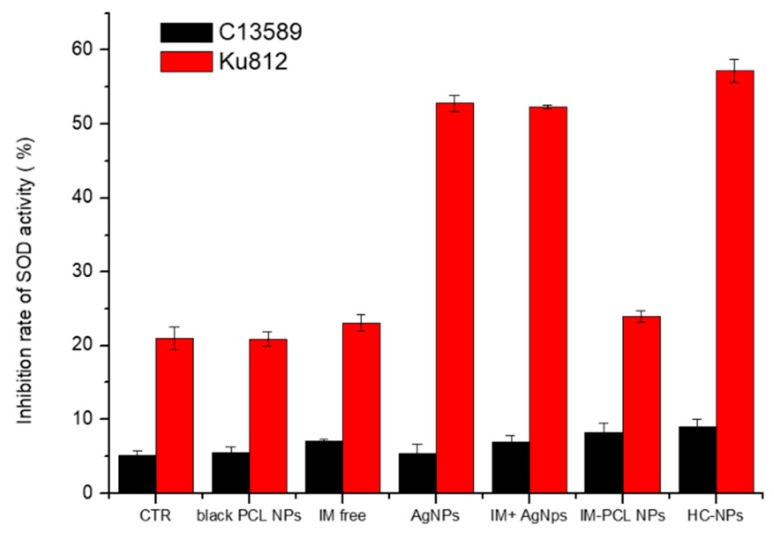
Inhibition rate of superoxide dismutase activity (SOD) in leukaemia KU812 and normal C13589 cell line after 6 h of treatment with black PCL NPs, free imatinib mesylate (IM), AgNPs, free IM/AgNP combination, IM-PCL NPs, and HC-NPs, compared with untreated control cells (CTR). Representative measurements of three independent experiments have been reported (*P* 0.05).

In addition, we have validated the production of by reactive oxygen intermediates (ROI) metabolites that determinate cell death through the activity of superoxide dismutase (SOD). The inhibition rate of superoxide dismutase activity was significantly increased in HC-NP-treated KU812 cells at LD_50_ concentrations, compared with untreated control cells (CTR) and normal C13589 cell line, as show in Figure 7.

## 3. Discussion

Side effects, such as infections, are often present after chemotherapy. However, this challenge can be overcome by performing targeted combination therapy, using a low dose of chemotherapic drugs and antimicrobial agents. Multifunctional nano-systems can be used to combine chemo and antibacterial agents in one single nano-system; in this way, is possible to specifically kill cancer cells and prevent infections.

Different studies have described the combination of different therapeutic agents inside one NP [13,14] to overcome the drug resistance of cancer cells [27,28]. No studies in the literature, however, report the combination in one single nanosystem of therapeutic agents with anticancer and antimicrobial properties.

In this work, we report a nano-platform in which a chemotherapic drug (IM) is combined with silver, showing good cancer action using a low dose and a good antibacterial effect. 

Over the last few years, we have developed enzymatic [24,29] and pH-responsive polymeric [30] NPs for drug therapy in leukemia. Recently, we have reported [10] the effect of two different drugs combined into one single nanosystem, which showed the potentiality of overcoming drug resistance with low concentrations in a synergic effect.

In this paper, we adopted a different strategy by developing hybrid clustered nanoparticles (HC-NPs) to combine both the chemo/antibacterial potential of silver nanoparticles and therapeutic advantages of IM drugs in order to reduce adverse effects, such infections, and to achieve a cancer cure.

The onion-like structure of our HC-NPs was formed specifically by two different nanoparticles. The inner core structure was characterized by a mothership nanocarrier (size of approximately 200 nm) with the role of compartmentalized loaded payload polymeric nanovector, functionalized with specific monoclonal antibody (mAb), in order to specifically target cancer. The external surface of the mothership was covered with 30 nm-sized babyship silver nanocarriers to induce anticancer and antibacterial effects. With this HC-NP configuration, we expected a multistep release mechanism (see scheme in Supplementary Figure S11) in which (i) HC-NPs were internalized by target cells thanks to the specific antibody and degradation of enzyme-sensible layer of PRM by intracellular proteases; (ii) the acidification of the capsosoma environment allowed the release of babyship NPs complexed with pH-sensible polymers (first release) into the cytoplasm. In particular, an initial babyship AgNP release started in the cytoplasm, thanks to the acidification of the capsosoma environment, and their complete release occurred in the endosome/lysosome compartment; and (iii) and (iv) babyship NPs and mothership NPs (inner core) were carried into the lysosomal compartment. In this environment (pH 5.0) the generation of CO_2_ bubbles and the cationization of the mothership NPs led to their escape into the cytoplasmic compartment and the release of IM (second release); on the other hand, babyship AgNPs released Ag^+^ ions and allowed ROS production. The release of IM and ROS production by Ag^+^ ions facilitated the killing of leukemia cells (cancer therapy) and, at the same time, the release by dying cells (see schema in Supplementary Figure S11, point (v) of AgNPs and Ag^+^ ions, which allowed for antibacterial activity external of the cancer cell. 

A good nanosystem for cancer therapy should have a high loading efficiency. In our study, the IM encapsulation efficiency (EE%) obtained was about 95% in PCL NPs and 73% for AgNPs. The build-up of different layers on the mothership NPs did not influence the IM EE%. 

In addition, the release study (Supplementary Figure S5) of babyship AgNPs and IM by HC-NPs showed a different release when the HC-NPs were incubated in a solution of PBS with different pH values (5.8 and 7.4) that mimicked lysosomal and cytoplasmatic milieus. The release of IM was subjected at the protease degradation of DXS, and the release was different pH values after 96 h (about 42%). On the contrary, we detected an initial rapid release in the first hours and a gradual and sustained release in the late time of AgNPs at pH 5.8 (about 81%). At pH 7.4, we observed a low release for AgNPs (only 5%). This dissimilar behavior can be due to the fact that during synthesis process, in the NP core, a mixture of sodium bicarbonate and potassium tartrate was added, which swiftly produced CO_2_ bubbles in the acidic pH in order to produce holes in the NP’s shell and support IM release [10]. In an acid compartment of lyso/endosomal (pH 5.8) the sodium bicarbonate and potassium tartrate combination rapidly produced CO_2_ bubbles, allowing the formation of big pores in the NP wall, which allowed the release of payloads, as demonstrated in our previous work [10]. The release profiles of IM and AgNPs showed that our HC-NPs responded specifically to the environmental conditions (pH and proteases) for which they were produced. In addition, as we have demonstrated, the inner core presented a negative charge in physiological pH, which was reverted to a positive charge in the acidic pH of endo-lysosomal vesicles [10,31], determining NPs endo-lysosomal escape.

Another important aspect to consider is the fact that in a physiological environment, NPs interact with different biomolecules and form a protein corona [32,33], which affects the cellular response and the recognition by mononuclear phagocyte system. It is reported that a low quantity of proteins that form the protein corona around nanoparticles induce a reduction of macrophages uptake [34]. We have observed that AgNPs and PCL NPs present a high quantity of protein corona after incubation with complete medium or mouse blood plasma (Figure 2) when compared with HC-NPs. This dissimilar protein absorption was attributed to the modified surface of HC-NPs that reduced the protein corona. A lower protein corona arrangement can influence the in vivo destiny of HC-NPs via immune elusion and short uptake by mononuclear phagocytic system. 

An additional key parameter that can influence the possibility of use of NPs in vivo is hemocompatibility. After incubation with mouse blood (Supplementary Figure S6), the HC-NPs’ hemolytic rate was lower compared to the positive control (physiological solution and blood). Our results confirmed that our HC-NPs were hemocompatible with mouse erythrocytes, and that they can be administrated intravenously.

The key point of this study was the combination of organic and inorganic NPs inside one single nano-platform. Inorganic NPs [15], such as AgNPs with significant cancer [14,16,17] and antimicrobial properties [21], were combined in our HC-NP platform. In particular, the FDA has approved the human use of AgNPs as an antimicrobial agent [21], and their antibacterial properties have been studied extensively [35,36]. In this context, we have designed our HC-NPs in order to prevent the infections in patients after chemotherapy treatments, and AgNPs result as a powerful broad-spectrum antibacterial agent. AgNPs and HC-NPs have shown a good antibacterial capacity on Gram-negative *E. coli*, DH5α *E. coli*, and Gram-positive *S. aureus* (Figure 3), confirming the antibacterial efficacy of the babyship AgNPs inside the HC-NPs, which also can be used as a prophylaxis tool.

In this study, we chose chronic myeloid leukemia cells as a disease model for validation of our HC-NPs. Clinical treatment of this disease makes use of tyrosin kinase inhibitors, such as IM. This drug is active against BCR-ABL oncoprotein expressed by this cell population. It is renown, in fact, that IM is cytostatic and not cytotoxic in the CML stem cell compartment, and for this reason, we loaded the IM into NPs along with AgNPs with anticancer properties, as the use IM alone is unable to remove leukemic stem cells. 

Cell targeting is a crucial step for specific delivery of active molecules to cancer cells, in order to avoid an adverse effect on healthy cells. For this aim, we have functionalized our HC-NPs with an antibody against CD38 specific for leukemia cells. We investigated HC-NP-specific cell targeting using a co-culture of CD38-positive (KU812) and CD38-negative (C13895) cells for 24 h. Confocal analysis confirmed that HC-NPs can specifically target leukemia KU812 cells in a co-culture (Figure 4) and the specific targeting established the importance of the antibody anti-CD38 functionalization of our HC-NPs, in order to improve the efficacy of therapy and to reduce side effects. 

Combinatorial cancer therapy using a combination of drugs inside one nanosystem has revealed an enhanced chemotherapy efficacy, reducing toxic side effects [10,13,14]. In addition, AgNPs have important cancer activity [14,16,17] and no adverse effect on healthy cells [17,37]. In our HC-NPs, we combined the specific anticancer activity of IM that binds specifically to the BCR-ABL oncoprotein present only in the CML cells, and the anticancer property of AgNPs, thanks to the production of ROS, in order to improve their efficacy on cancer cells only.

Using different NP formulations, we investigated the anti-cancer effects of HC-NPs in vitro. No cytotoxic effects were observed on healthy C13895 cells using all formulations of the IC_50_ doses (Supplementary Figure S8). Healthy cells did not respond to IM because of the absence of the oncoprotein BCR-ABL. In addition, in healthy cells, the AgNPs did not induce cytotoxicity because these present a functional antioxidant system. A dose-dependent effect (Supplementary Figure S10A) was observed when leukemia cells were incubated with all NP formulations (IM loaded PCL NPs, AgNPs, and HC-NPs). Specifically, HC-NP formulation with a lower IC_50_ value showed an enhanced cytotoxic effect for HC-NPs due the controlled release of the drug and the AgNPs. Moreover, an advanced synergistic effect with a combination index (CI) of 0.81 of IM and AgNPs on KU812 leukemia cells was observed. In vitro cytotoxicity showed that the combination of IM and AgNPs induced an improved anti-leukemia effect at low concentrations. 

The combinatorial effect on leukemia cells (Figure 5A) was more evident when incubated with HC-NPs (about 72%) with high apoptosis. Also, cell cycle blocking at the G2/M phase (see Figure 5B) was noticed when treated with HC-NPs (~45%). In vitro studies indicated that the combinatory effect of our HC-NPs exhibited an excellent anti-leukemic activity, improving cell death efficiency.

The long-time inhibition of tyrosine kinase activity of BCR-ABL in leukemic cells KU812 was evaluated by western blotting analysis after 7 days, using different formulations. As shown in Figure 6, a sustained and total inhibition of BCR-ABL was evident when leukemia cells were treated with HC-NPs. It is important to note that BCR-ABL tyrosine phosphorylation was only prevented using free IM or in free combination with AgNPs, whereas the co-encapsulation of the IM and AgNPs in HC-NPs achieved an effective in vitro inhibition. Improving the inhibition BCR-ABL tyrosine activity for a long-time window will possibly allow an extended block of BCR-ABL autokinase action that is decisive in endorsing cell apoptosis.

Moreover, the production of reactive oxygen intermediate (ROI) metabolites inducted by AgNPs, which determinate cell death, was investigated through the activity of superoxide dismutase (SOD), showing a significant increase of the inhibition rate of superoxide dismutase activity in KU812 cells treated with HC-NPs at LD_50_ concentrations, as shown in Figure 7. In healthy C13895 cells with an efficient antioxidant system, no increasing of SOD was observed.

The toxic effect of different formulation of NPs on the appearance and the general behavioral pattern of healthy mice was evaluated in preliminary experiments. No toxic symptoms or mortality were observed in any animal. Similarly, at a general observation, no changes in behavioral pattern, clinical signs, or food consumption were noticed in both control and treated mice (data not shown). In vivo experiments treating leukemia xenograft mice are the subject of future work.

In this work, a chemo-antibacterial combinatorial therapy strategy was developed, which potentiated single drugs and elicited an antibacterial response. Our strategy may have important therapeutic and pharmacological applications in cancer therapy, in order to simultaneously kill cancer cells and prevent infections.

## 4. Materials and Methods

Media for cell culture and chemicals were acquired from Sigma-Aldrich. Human chronic myeloid leukaemia cells (KU812), human normal B lymphoblast (C13589), and Gram-negative DH5-Alpha *E. coli* were acquired from the American Tissue Type Collection (ATTC). Gram-positive *S. aureus* was obtained from Dr. Federica Paladini. Annexin V-PI kit and nitrocellulose membrane were obtained from Abcam; Coomassie brilliant blue staining and Clarity—Western ECL Substrate were obtained from BioRad; anti-c-ABL antibody was obtained from Santa Cruz Biotechnology Inc.; HRP-conjugated antibody was obtained from Cell Signaling.

Blood for protein corona analysis and hemolytic activity was collected from male adult C57BL/6j mice, weighting 20–30 g. For protein corona analysis, blood was collected in tubes with heparin and centrifugated in order to obtain the plasma. All procedures involving animal care were approved by the ethics committee of La Sapienza University (Rome), the Animal Care and Use Committee of the Italian Ministry of Health, and performed in compliance with the guidelines of the European Community Council (2010/63/UE) and the decree law (D.L.) 26/2014 of Italian Ministry of Health. All efforts were made to minimize animal suffering and to reduce the number of animals used.

### 4.1. Synthesis and Characterization of AgNPs

A 50 mL aqueous solution containing AgNO_3_ (1 mM) was kept under constant agitation on a magnetic stirrer at 1500 rpm in ice and mixed with an equal volume of an aqueous solution of sodium citrate (50 mM). Subsequently, 75 mL of NaBH_4_ (20 mM) was added drop by drop into this solution until it became yellow. Next, the AgNP suspension was washed three times with water by centrifugation at 12,500 rpm for 20 min and then resuspended in sodium citrate and stored at 4 °C until use.

### 4.2. Synthesis of HC-NPs

#### 4.2.1. Synthesis of IM Loaded PCL NPs

Poly ε- caprolactone (PCL) NPs loaded with IM were synthetized, as according to our previous works [10]. A homogenous size of NPs, density gradient centrifugation was applied using 5, 10, 15, 20, and 25% w/v sucrose dissolved in PBS 1×. In an Eppendorf tube, 200 μL of each sucrose solution were layered one on the top of each other. One-hundred microliters of NPs solution was layered on the top of the sucrose layer and was centrifuged at 14,000 rpm for 60 min. After centrifugation, 10 μL from each layer was analyzed by DLS. 

#### 4.2.2. Synthesis of HC-NPs

Hybrid IM-PCL/Ag cluster NPs (HC-NPs) were obtained by coating with the layer-by-layer technique (LbL), with the IM loaded PCL NPs (1 mg) using 1 mL of chitosan medium molecular weight (CH, 3 mg/mL in NaCl 0.1M). The dispersion was continuously shaken for 10 min. The excess of CH was removed by three centrifugation/washing steps with a 0.1 M NaCl solution. Thereafter, 1 mL of AgNPs (0.1 to 10 ppm) was added, and the dispersion was continuously shaken for 2 h, followed again by three centrifugations (12,500 rpm for 20 min) and washing steps. Subsequently, 1 mL of a 0.1 M NaCl solution containing the polycation protamine (PRM, 2 mg/mL) was added, and the dispersion was continuously shaken for 10 min, followed again by three centrifugation/washing steps. The mixture was then dialyzed with pure water for 8 h. For fluorescent HC-NPs, a 0.05 mg/mL of DiOC_18_(3)(3,3’-Dioctadecyloxacarbocyanine Perchlorate), DiO, in chloroform was added to the PCL solution and the preparation was carried out as described previously [10]. The labelled HC-NPs were stored in the dark at 4 °C until use. 

#### 4.2.3. HC-NPs Functionalization with Anti-CD38 Antibody

For covalent binding of monoclonal anti-CD38 antibody onto HC-NPs, 5 μg of EDC (1-ethyl-3-(3-dimethylaminopropyl)-carbodiimide) was mixed with 400 μL of a solution containing 450 μL of HC-NPs and 450 μL of antibody; in this way, the molar ratio between EDC and antibody was about 9. The mixed solution was stirred under constant agitation on a magnetic stirrer at 500 rpm for 2 h at room temperature (RT). After incubation, the sample was centrifuged at 12,500 rpm for 20 min, and the pellet was washed three times with 1 mL of blocking and storage buffer (0.1 M boric acid and 0.1% Bovine Serum Albumin (BSA), pH 7.5) containing 2 μL of TRITC-conjugated secondary antibody (1:250). The sample was stirred gently for 1 h at RT and was then centrifuged, and the pellet of the HC-NP antibody conjugate was suspended in 1 mL of blocking and storage buffer and kept in the dark at 4 °C until use.

### 4.3. Characterization of NPs

#### 4.3.1. Dynamic Light Scattering 

Hydrodynamic size and zeta potential of AgNPs, IM-PCL NPs, and HC-NPs were evaluated by a Zetasizer Nano ZS90 (Malvern Instruments Ltd, USA). Stability of HC-NPs over time (8 days) in complete Roswell Park Memorial Institute (RPMI) 1640 medium was verified by DLS analysis (three independent experiments).

Fixed aqueous layer thickness (FALT) was calculated on the basis of zeta potential of NP solutions with a serial dilution with an isotonic solution of 10 mM lactate buffer (pH 4) with various concentration of NaCl and sucrose. The ln zeta potential ζ (V) was plotted against the Debye Hϋckel parameter (k). k represents 3.3vC, where C is the concentration of electrolytes in the solution. The slope of the obtained plots indicated the thickness of the fixed aqueous layer in nm. 

#### 4.3.2. UV-VIS Spectroscopy 

UV-visible spectra were acquired with a UV-visible spectrophotometer (Varian Cary 300 Scan; Varian Instruments, CA, USA) at RT, and spectral analysis was performed in the 300–800 nm range.

#### 4.3.3. Scanning and Transmission Electron Microscopy 

The external morphology of AgNPs, IM-PCL NPs, and HC-NPs was examined by scanning electron microscopy (SEM) and transmission electron microscopy (TEM). Prior to SEM analysis, the samples were coated with a 10 nm gold layer. SEM analyses were taken with a Carl Zeiss Merlin SEM supplied with a Gemini II column and a field emission gun (FEG). In addition, AgNPs were analyzed using a JEOL Jem 1011 TEM microscope (Japan).

#### 4.3.4. FT-IR Spectroscopy 

FT-IR analysis was performed using a VERTEX 70v FT-IR Spectrometer (Bruker) in order to assess the secondary structure of AgNPs, PCL NPs, and HC-NPs. Infrared (IR) spectra were acquired in absorbance mode, and each spectrum was obtained by the 60 scans with the wavenumber ranging from 0 to 4000 cm^−1^.

#### 4.3.5. Flow Cytometry and SDS-PAGE Assay 

To confirm the antibody functionalization on the HC-NPs’ surface, we performed a flow cytometry analysis. One milliliter of HC-NPs conjugated with anti-CD38 antibody labelled with TRITC anti-mouse secondary antibody was introduced into a flow cytometer and analyzed using a C6 Flow Cytometer (Accuri, USA). As a control, non-conjugated HC-NPs were used. The antibody content onto HC-NPs was determined by bicinchoninic acid (BCA) protein assay according to the manufacturer’s instructions (Sigma-Aldrich, USA). The BSA was used as a standard and the antibody conjugated was quantified using a UV-visible spectrophotometer (Varian Cary 300 Scan; Varian Instruments, CA, USA) at a wavelength of 562 nm. In addition, to control the integrity of antibody after conjugation, HC-NPs were subjected to 4%–12% SDS-polyacrylamide gel electrophoresis (SDS-PAGE). The resolved protein bands were visualized by Coomassie brilliant blue staining (BioRad), according to the manufacturer’s instructions. Representative results of three independent experiments were reported.

#### 4.3.6. Protein Corona 

Protein corona analyses were achieved by SDS-PAGE. First, 500 ng/mL of AgNPs, PCL NPs, and HC-NPs were raised in complete RPMI medium supplemented with 10% FBS or with 2% of mice blood plasma at 37 °C overnight in a shaker. Afterward, NPs were centrifuged in order to remove hard corona and resuspended in PBS 1×. Washing procedure was repeated three times prior to SDS-PAGE. Protein concentration on NPs was determined by using bicinchoninic acid (BCA, Sigma-Aldrich) at 562 nm. Eluted hard corona proteins from all samples were mixed with SDS sample buffer and boiled at 100 °C for 10 min. The samples were subjected to 4–12% SDS-PAGE for 90 min at 120 V. Coomassie blue staining was used for detection protein bands. 

In addition, zeta potential was measured by DLS analysis.

Representative results of three independent experiments have been reported.

#### 4.3.7. Hemolytic Activity

Hemolysis assays were performed incubating the HC-NPs with 2% mouse blood suspension for 24 h at 37 °C. After the suspension, it was centrifuged at 15,000 rpm for 20 min at 4 °C, and the supernatant adsorption (A) was analyzed by UV-visible spectrophotometer (Varian Cary 300 Scan; Varian Instruments, CA, USA) at a wavelength of 540 nm. Hemolytic rate was calculated using the following equation:

$$\mathrm{Hemolytic}\;\mathrm{rate}\;\left(\%\right)=\frac{A\left(\mathrm{material}\right)-A\left(\mathrm{negative}\;\mathrm{control}\right)}{A\left(\mathrm{positive}\;\mathrm{control}\right)-A\left(\mathrm{negative}\;\mathrm{control}\right)}\times 100$$

where negative control was composed of saline solution and blood without NPs, while positive control was saline solution. Representative results of three independent experiments were reported.

Antibacterial activity. Gram-negative DH5-Alpha *E. coli* and Gram-positive *S. aureus* were grown in a Luria–Bertani (LB) broth. For antibacterial assay, growth bacterial inoculum (10^4^ cells/mL) was incubated with different formulation of NPs, such as AgNPs and HC-NPs. Optical density (OD) at a wavelength of 600 nm using a UV-visible spectrophotometer (Varian Cary 300 Scan; Varian Instruments, CA, USA) every 1 h to measure the bacteria growth. In addition, Gram-negative DH5-Alpha *E. coli* and Gram-positive *S. aureus* treated with different NP formulation were spread on solid LB plates, and the colony forming unit number (CFU) was counted using a Miles–Misra method after 24 h at 37 °C. Representative measurements of three different sets of data were reported.

Entrapment efficacy and in vitro release. The IM entrapment efficacy of mothership PCL NPs and babyship AgNPs into HC-NPs was evaluated as described in [10], through analysis of the supernatant after the NPs’ centrifugation.

### 4.4. Targeting and HC-NP Cellular Uptake

KU812 leukemia cells and C13589 normal lymphoblasts were maintained in culture using RPMI 1640 medium with 10% FBS. To study the ability of mAb-conjugated HC-NPs to target specific cells, a co-culture of CD38-positive (KU812) and CD38-negative (C13895) cells was prepared by seeding 10^4^ CSFE labelled C13589 with 10^4^ KU812 cells and treated with 500 ng/mL of TRITC-conjugated anti-CD38 HC-NPs for 24 h. For fluorescence confocal analysis, cells were fixed for 5 min in 3.7% formaldehyde and mounted. 

Cellular uptake efficiency (%) of HC-NPs was evaluated in accordance with [10]. Representative measurements of three distinct sets of data have been reported. 

### 4.5. In Vitro Cancer Efficacy 

In vitro cytotoxicity of different formulations was analyzed using an MTT test after 2 days of treatment, and the IC_50_ was calculated.

IM and AgNPs’ synergistic effect were calculated using a combination index (CI) [38].

#### 4.5.1. Apoptosis and Cell Cycle Analysis

Apoptosis and cell cycle were examined with annexin V-FITC/PI assay, and 10,000 ungated cells were evaluated with a Flow Cytometer (C6, Accuri, USA). In particular, KU812 cells (10^5^ cells/mL) were incubated with the different formulations, such as 500 ng/mL of empty PCL NPs, 150 nM of IM, 250 nM of AgNPs, 100 nM of IM/AgNPs free combination in the medium, 100 nM of IM-PCL NPs, and 50 nM of HC-NPs for 24 h. All experiments were performed in triplicate.

#### 4.5.2. Western Blotting for BCR-ABL Inhibition Analysis

KU812 leukemia cells (10^6^ cells/mL) were incubated with 500 ng/mL of empty PCL NPs, 150 nM of IM, 250 nM of AgNPs, 100 nM of IM/AgNPs free combination in the medium, 100 nM of IM-PCL NPs, and 50 nM of HC-NPs. After 7 days of incubations with the different formulations, the samples were washed in PBS 1× at 4 °C and resuspended in lysis buffer containing a protease inhibitor cocktail on ice for 30 min. Protein concentration was determined using the BCA protein assay (Sigma-Aldrich). Protein bands were separated on 10% (w/v) SDS-polyacrylamide gels, and immunoblotting was performed using nitrocellulose membrane (Amersham Hybond ECL Nitrocellulose Membrane-GE, Abcam). Primary incubations with specific primary antibody directed against anti-c-ABL 1:1000 (clone K-12, Santa Cruz Biotechnology Inc., CA, USA) and anti-βactin 1:5000 (Sigma-Aldrich) were performed overnight. Secondary incubations were for 1 h with HRP-conjugated anti-mouse antibody (Cell Signaling). Proteins were visualized by chemiluminescence (Clarity—Western ECL Substrate, BioRad) using the C-DiGit blot scanner (LI-COR, Cornaredo Milano, Italy). Densitometric analysis was performed using Image J software on the western blots, normalizing to β-actin used as control.

#### 4.5.3. Superoxide Dismutase (SOD) Assay

Cell death can be produced by reactive oxygen intermediates (ROI). Superoxide dismutase (SOD), which catalyzes the dismutation of the superoxide anion (O_2_) into hydrogen peroxide and molecular oxygen, is one of the most important antioxidative enzymes. Antioxidant production was measured using a superoxide dismutase (SOD) assay kit (Sigma-Aldrich, USA) according to the manufacturer’s instructions. Briefly, to determine the activity of SOD, human chronic leukemia cells (KU812) and normal human B lymphocyte cells (C13589) were incubated with blank PCL NPs (500 ng/mL), free IM (150 nM), AgNPs (250 nM), free combination of IM/AgNPs (100 nM), IM-PCL NPs (100 nM), and HC-NPs (50 nM) for 6 h. Cells were then washed three times with PBS and sonicated on ice in an ultrasonicator (80 watts outpower) for 15 s periods for a total of 4 min. The solution was then centrifuged at 1500 rpm for 5 min at 4°C. The resulting supernatants were used to determine intracellular antioxidants using a spectrophotometer at 440 nm. Each assay was performed in triplicate.

### 4.6. Statistical Analysis

Three independent experiments were performed, and the results are expressed as mean ± standard deviation. Statistical analysis was achieved by one-way ANOVA.

## 5. Conclusions

In this study, a hybrid clustered nanoparticle that serially responds to endogenous stimuli was developed for chemo-antibacterial combinatorial cancer therapy.

In vitro studies showed an excellent anti-cancer activity of HC-NPs on chronic myeloid leukemia (CML), used as a model of cancer, improving the cell death efficacy thanks to the combinatory effect of chemotherapic drug and AgNPs. In addition, our HC-NPs showed a good antibacterial capacity on Gram-negative and Gram-positive bacteria.

The chemo-antibacterial combinatorial therapy strategy developed in this study, which potentiates the presence of single drugs and elicits an antibacterial response, paves the way for developing new multifunctional nanoplatforms in cancer therapy.

## Figures and Tables

**Table 1:** Physicochemical characterization of different nanoparticle (NP) formulations. Representative measurements of three different sets of data have been reported (*P*-values 0.05). HC-NPs: hybrid clustered nanoparticles, FALT: fixed aqueous layer thickness, PdI: Polydispersion Index.

Sample	ζ Potential (mV)	Size (nm)	PdI	FALT (nm)
Ag NPs	−27.9 ± 0.96	32.02 ± 0.69	0.6 ± 0.06	-
PCL NPs	−11.5 ± 0.24	228.1 ± 0.3	0.36 ± 0.03	-
PCL-Ag NPs	−25.4 ± 0.15	274.8 ± 0.25	0.57 ± 0.01	1.82 ± 0.02
HC-NPs	10.8 ± 0.88	286.5 ± 0.56	0.62 ± 0.05	2.55 ± 0.03

## Supplementary Materials

The following are available online at https://www.mdpi.com/2072-6694/11/9/1338/s1, Figure S1. Absorption spectra of AgNPs. Figure S2. TEM image of AgNPs and SEM images of black PCL NPs, PCL-Ag NPs, and HC-NPs. Figure S3. FT-IR analysis of AgNPs, PCL NPs, and HC-NPs. Figure S4. FACS analysis and SDS-PAGE of non-conjugated and mAb-coated HC-NPs. Figure S5. In vitro IM and AgNPs’ cumulative release from HC-NPs. Figure S6. Hemolysis assay (HR %) of AgNPs, PCL NPs, and HC-NPs after 24 h. Figure S7. Time-dependent cellular uptake efficiency of not conjugated HC-NPs with antibody. Figure S8. Cytotoxicity of free IM, AgNPs, free IM/AgNPs combination, IM-PCL NPs, and HC-NPs after 48 h toward C13895 healthy cells. Figure S9. Analysis of cell apoptosis and cell cycle of C13895 healthy cells after 24 h of treatment with different formulations.Figure S10. Cytotoxicity assay using different NP formulations in KU812 cells after 48 h of treatment. IC_50_ values of NPs in KU812 leukemia cells. Figure S11. Illustration of multistep release mechanism.
