# Supplementary material for: Hybrid Clustered Nanoparticles for Chemo-Antibacterial Combinatorial Cancer Therapy
Source: Cancers (Basel). 2019 Sep 10;11(9):1338. doi: 10.3390/cancers11091338 (PMC6769784; doi:10.3390/cancers11091338)
Supplement: Supplementary file 1 [file cancers-11-01338-s001.pdf]

Supporting Information

Hybrid clustered nanoparticles for chemo-antibacterial combinatorial cancer therapy

Barbara Cortese,[1] Stefania D'Amone[2], Mariangela Testini[2], Patrizia Ratano[1] and Ilaria Elena Palamà[2*]

[1]Nanotechnology Institute, CNR-NANOTEC, University La Sapienza, P.zle A. Moro, Rome, 00185 Italy; barbara.cortese@nanotec.cnr.it; patrizia.ratano@nanotec.cnr.it

[2]Nanotechnology Institute, CNR-NANOTEC, via Monteroni, Lecce, 73100, Italy; stefania.damone@nanotec.cnr.it; mariangela.testini@nanotec.cnr.it; ilaria.palama@nanotec.cnr.it

* Correspondence: ilaria.palama@nanotec.cnr.it

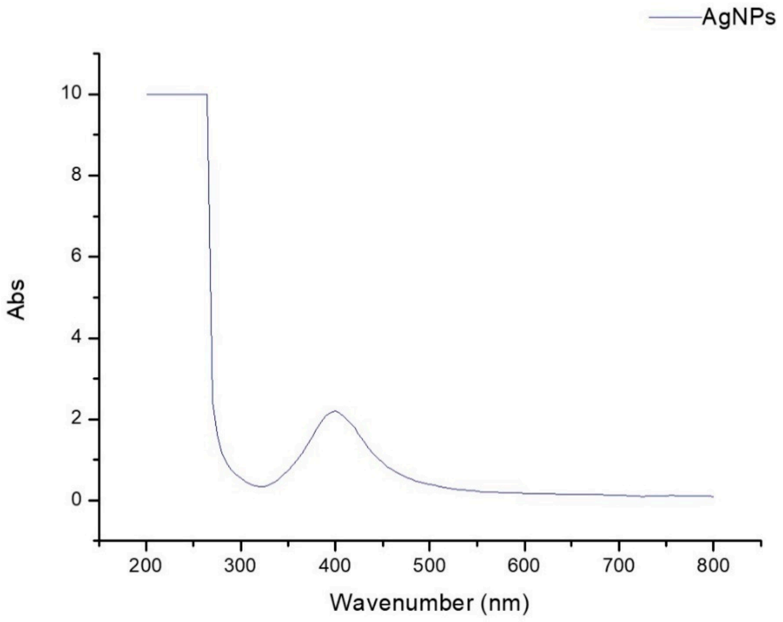

Figure S1. Absorption spectra of AgNPs. Representative measurements of three different sets of data have been reported.

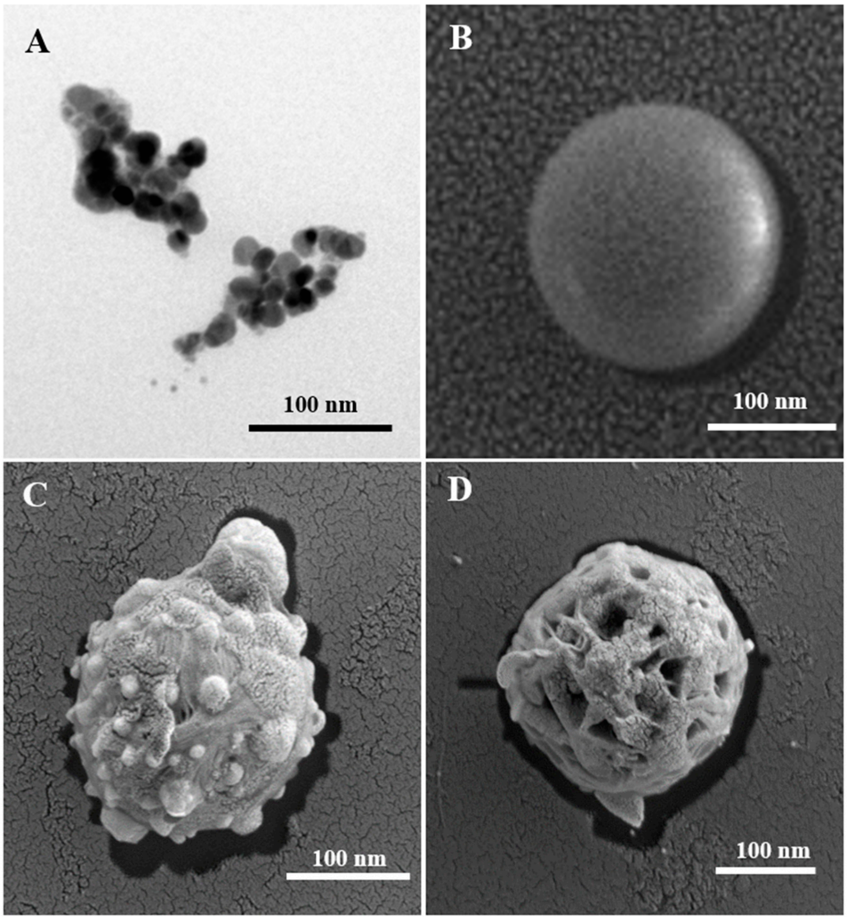

Figure S2. TEM image of AgNPs (A), SEM images of black PCL NPs (B), PCL-Ag NPs (C) and HC-NPs (D). *Scale bars: 100 nm*.

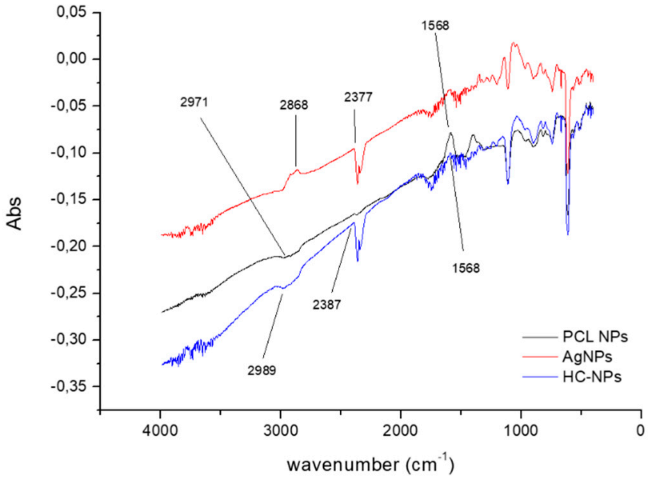

Figure S3. FT-IR analysis of AgNPs, PCL NPs and HC-NPs. Representative measurements of three different sets of data have been reported.

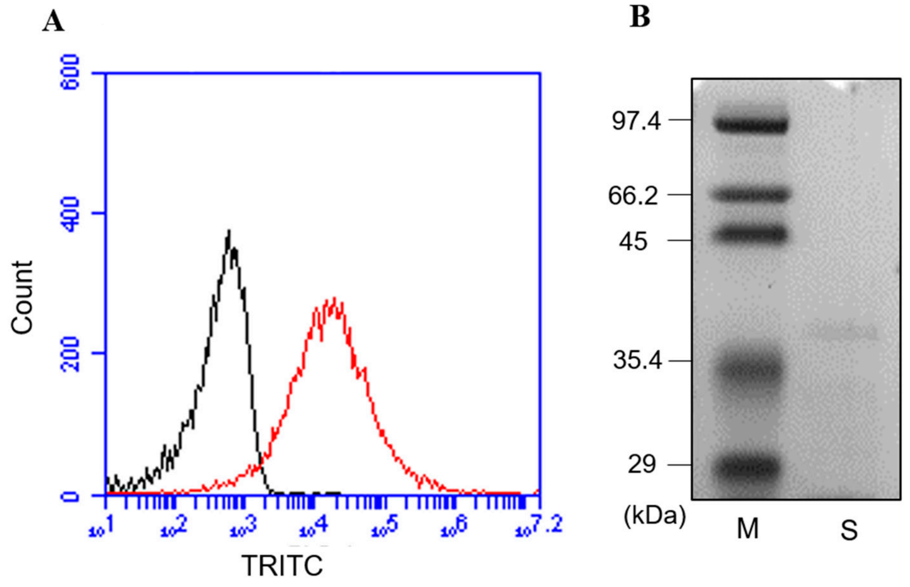

Figure S4. FACS analysis (A) of non-conjugated (black) and mAb coated HC-NPs (red). SDS-PAGE electrophoresis (B). Lane M, Marker; lane S, anti-CD38 antibody conjugated onto HC-NPs. A representative result of three independent experiments is shown.

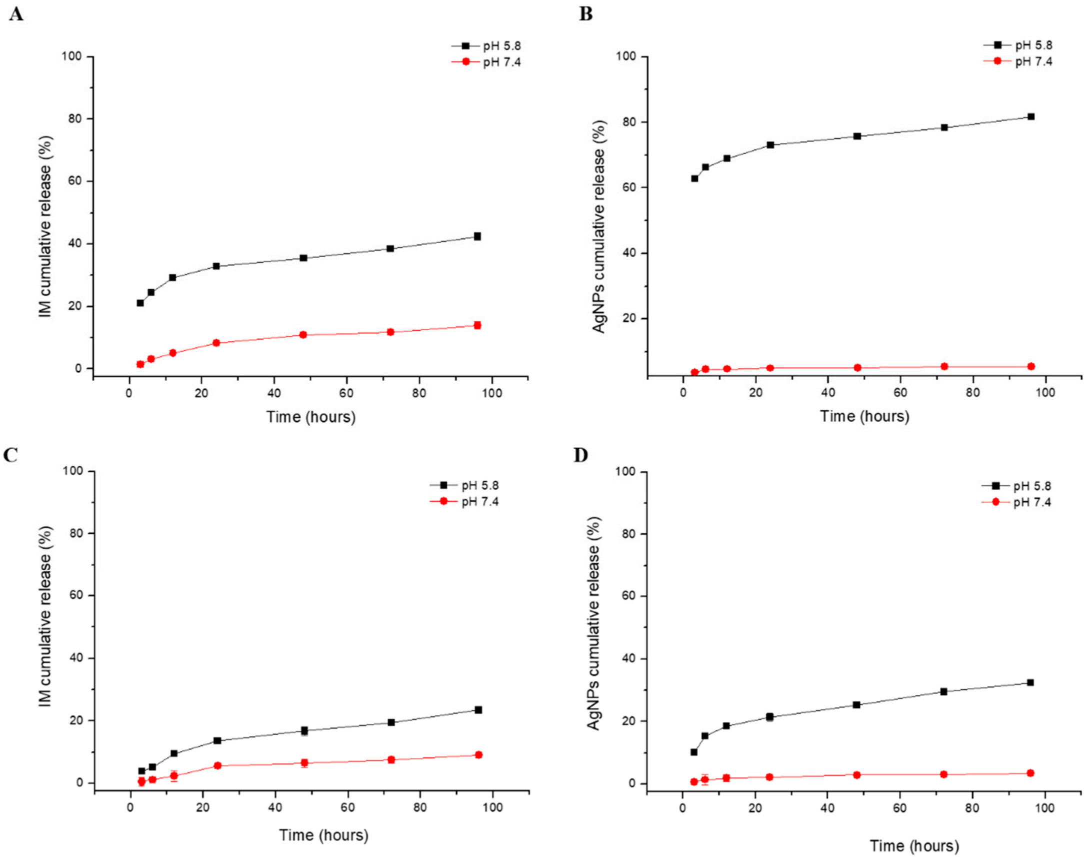

Figure S5. *In vitro* IM (A) and AgNPs (B) cumulative release from HC-NPs (formulation with the combination of sodium bicarbonate and potassium tartrate in the NPs core) at 37°C at

different pH. IM (C) and AgNPs (D) cumulative release from HC-NPs (formulation without the combination of sodium bicarbonate and potassium tartrate in the NPs core) at 37°C at different pH. Representative measurements of three different sets of data have been reported (P-values < 0.05).

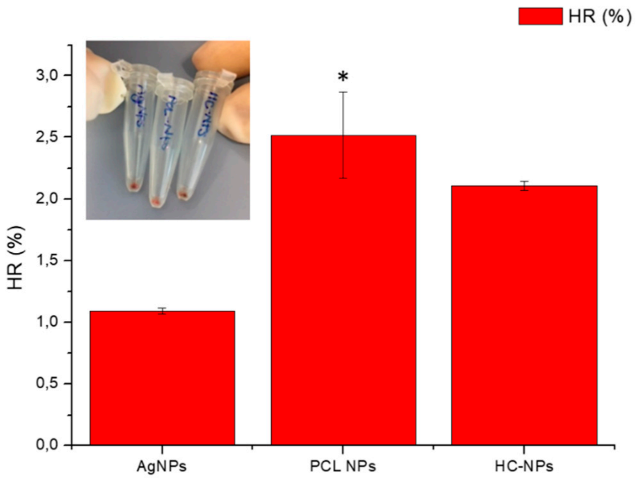

Figure S6. Hemolysis assay (HR %) of AgNPs, PCL NPs and HC-NPs after 24 hours and in the up-left inset is the photo of the hemolysis activity in different samples. Representative measurements of three independent experiments have been reported and * indicates *P-values < 0.05*.

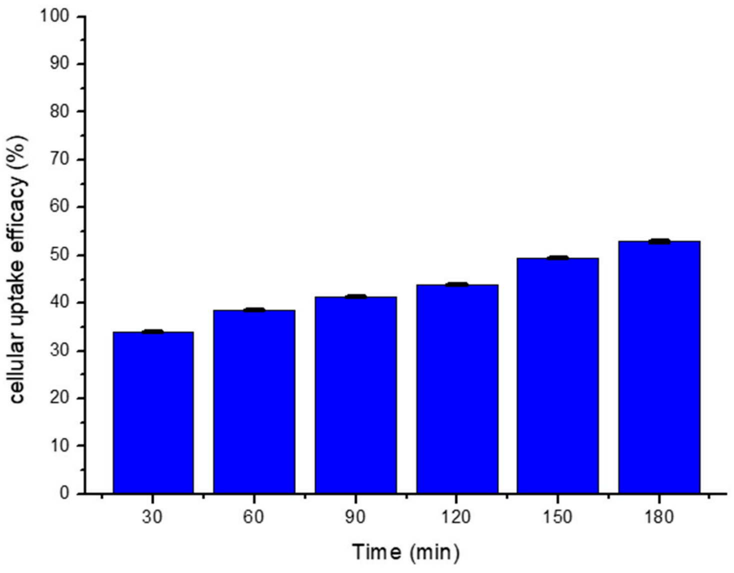

Figure S7. Time-dependent cellular uptake efficiency of not conjugated HC-NPs with anti-CD38 by KU812 cells. Representative measurements of three independent experiments have been reported (P-values < 0.05).

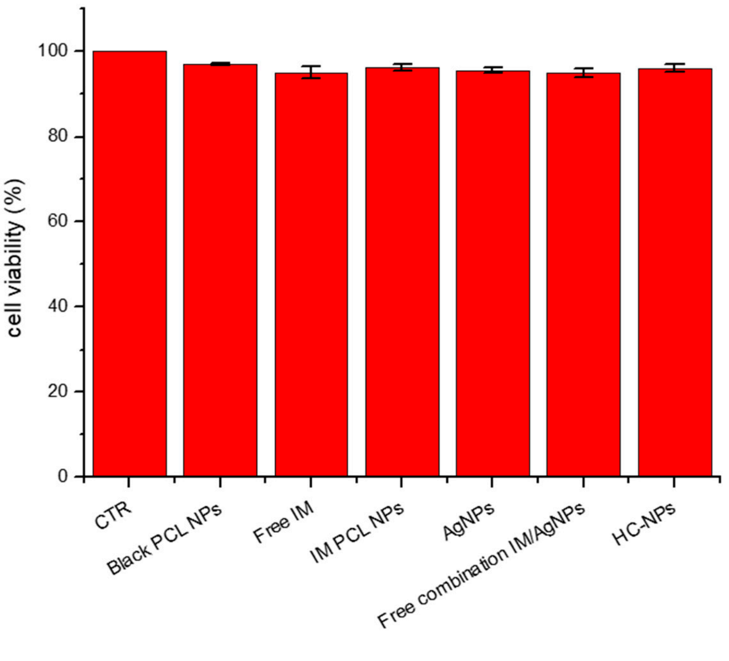

Figure S8. Cytotoxicity of free IM, AgNPs, free IM/AgNPs combination, IM-PCL NPs and HC-NPs after 48 hours toward C13895 healthy cells, compared with untreated control cells (CTR). Representative measurements of three independent experiments have been reported (P-values < 0.05)

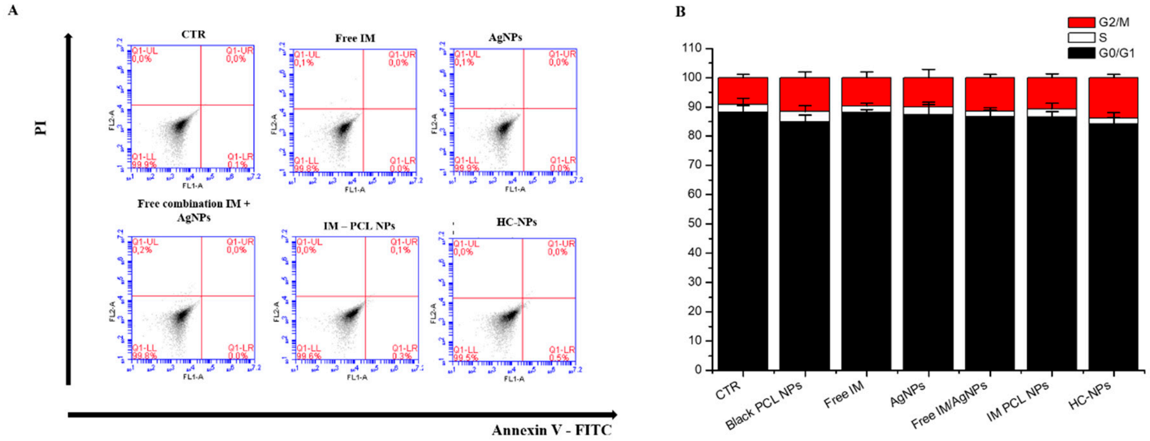

Figure S9. Analysis of cell apoptosis (A) and cell cycle (B) of C13895 healthy cells after 24 hours of treatment with different formulations, compared with untreated control cells (CTR). Representative measurements of three independent experiments have been reported ($P < 0.05$).

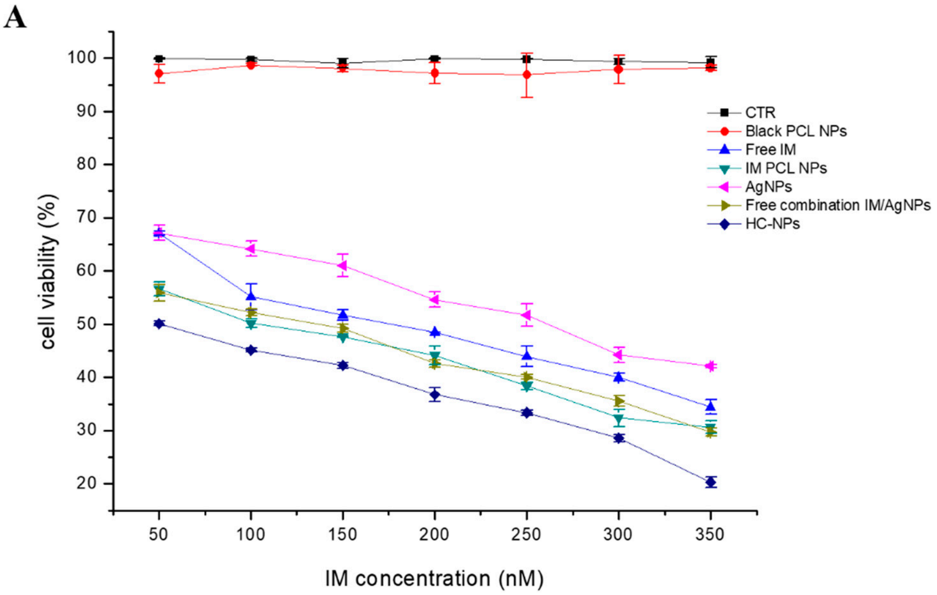

B

Sample	IC_{50} IM (nM)	IC_{50} Ag (nM)
Free IM	150 nM	-
IM released from PCL NPs	100 nM	-
AgNPs	-	250 nM
Free IM/AgNPs combination	130 nM	200 nM
HC-NPs	70 nM	180 nM

Figure S10. (A) Cytotoxicity assay using different NP formulations in KU812 cells after 48 hours of treatment. Untreated cells were used as control (CTR). Representative

measurements of three independent experiments have been reported (P-values < 0.05). (B) IC50 values of NPs in KU812 leukaemia cells.

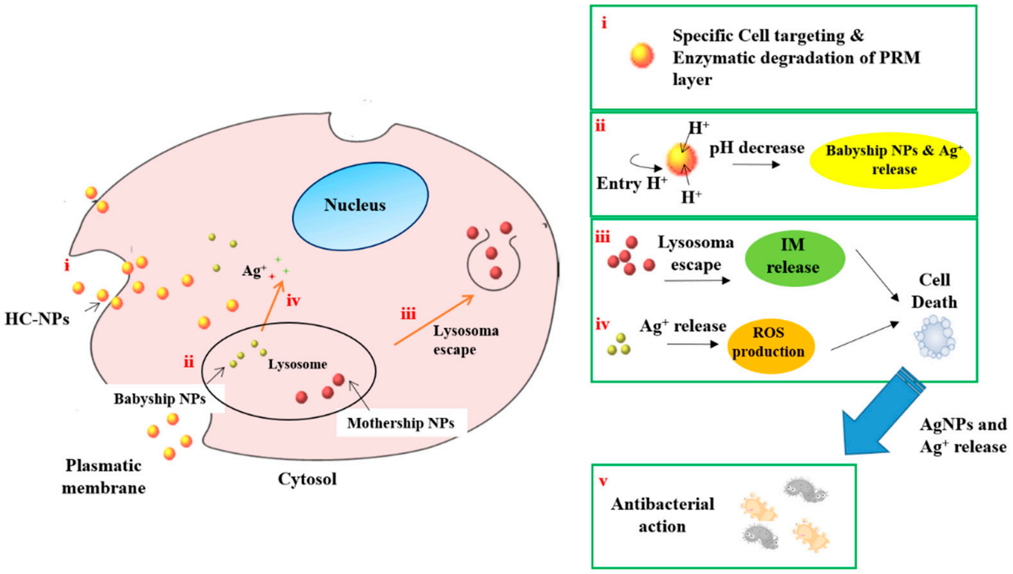

Figure S11. Illustration of multistep release mechanism.
